# A Review on Recent Advances in the Constitutive Modeling of Bone Tissue

**DOI:** 10.1007/s11914-020-00631-1

**Published:** 2020-10-17

**Authors:** Dieter H. Pahr, Andreas G. Reisinger

**Affiliations:** 1 Institute of Lightweight Design and Structural Biomechanics, TU-Wien, Vienna, Austria; 2 Department of Anatomy und Biomechanics, Karl Landsteiner University of Health Sciences, Krems, Austria

**Keywords:** Constitutive model, Material law, Bone tissue, Trabecular bone, Cortical bone, Review article, Finite element

## Abstract

**Purpose of Review:**

Image-based finite element analysis (FEA) to predict and understand the biomechanical response has become an essential methodology in musculoskeletal research. An important part of such simulation models is the constitutive material model of which recent advances are summarized in this review.

**Recent Findings:**

The review shows that existing models from other fields were introduced, such as cohesion zone (cortical bone) or phase-field models (trabecular bone). Some progress has been made in describing cortical bone involving physical mechanisms such as microcracks. Problems with validations at different length scales remain a problem.

**Summary:**

The improvement of recent constitutive models is partially obscured by uncertainties that affect overall predictions, such as image quality and calibration or boundary conditions. Nevertheless, in vivo CT-based FEA simulations based on a sophisticated constitutive behavior are a very valuable tool for clinical-related osteoporosis research.

## Introduction

### The Biological Material “Bone”

**Fig. 1:**
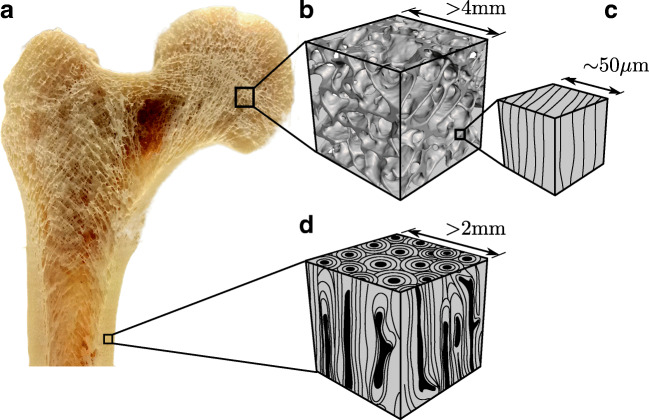
**a** Cross section of the human proximal femur showing the trabecular region and cortical shell. **b** Representative volume element (RVE) of the trabecular network and **c** RVE of bone tissue of single trabeculae. **d** Representative volume element of cortical bone. For the RVEs in **b**, **c**, **d**, constitutive material models are described in this manuscript

Bone is a living material, organized in a fascinating microstructural hierarchy [12, 37]. Besides its purpose as a mechanical support for the musculoskeletal system, it serves as a calcium reservoir [7], and a container for bone marrow. Bones in the human body consists of two distinct structures—the compact cortical shell and the spongy trabecular (or cancellous) region on the inside (Fig. 1). Cortical thickness ranges from several tenths of a millimeter to some millimeters in humans, while a trabecula is typically around 50–300 μm thick [37]. Individual trabeculae assemble to form foam-like structures with a typical bone volume fraction of 5–30% depending on anatomical location and age [48]. In cortical bone, Haversian and Volkmann channels, lacunae, and canaliculae lead to a cortical porosity of 4–17% depending on age [2, 3, 6].

The underlying microstructure of bone consists of lamellae of 3–6 μm thickness that arrange as osteons in compact bone and as lamellar packets in trabeculae [14, 35, 39]. Those lamellae are formed from bone fibrils—a highly aligned and organized staggered arrangement of bone’s basic constituents: collagen type I molecules, hydroxyapatite mineral platelets, water, and a small fraction of non-collagenous proteins [10, 50].

This complex hierarchical structure, alongside with the chemical composition, leads to the remarkable mechanical properties of bone that can be observed at different length scale levels. Pathological changes in nanostructural characteristics get manifest macroscopically, e.g., in osteogenesis imperfecta, where a collagen defect leads to brittle bones. This multi-scale interconnection of bone structures makes the constitutive modeling challenging.

### Mechanical Behavior of Bone

The response of biological tissues to mechanical loads has been the focus of biomechanical investigations for more than a century. On the material level, this response is described as the relationship between the size-independent measures of mechanical *stress* and *strain*. For a certain strain stimulus that is imposed, the material is reacting with stress response and vice versa. This response is intrinsic to the material and generally depends on the magnitude, the rate, the direction, the duration, and the number of repetitions of the imposed stimulus.

The description of an apparent behavior of an inhomogeneous material like bone requires an a priori definition of the considered length scale (nano, micro, macro). Microstructural features, like individual fibrils or lamellae, behave differently than bone at the tissue or organ level. For the sake of conciseness, this article will focus on the bone at higher length scales. One could imagine a representative cubical volume element (RVE) with a few millimeters in size, containing either cortical bone including vascular pores or a subsection of the trabecular network denoted as *trabecular bone* (Fig. 1b, d). Bone tissue properties at this macroscopic length scale are the most relevant in musculoskeletal research like implant technologies, tumors, trauma, and osteoporosis. Even at this scale, the description of the trabecular network relies on the underlying properties of the trabecular tissue, which is itself considered in an RVE as shown in Fig. 1c.

**Fig. 2:**
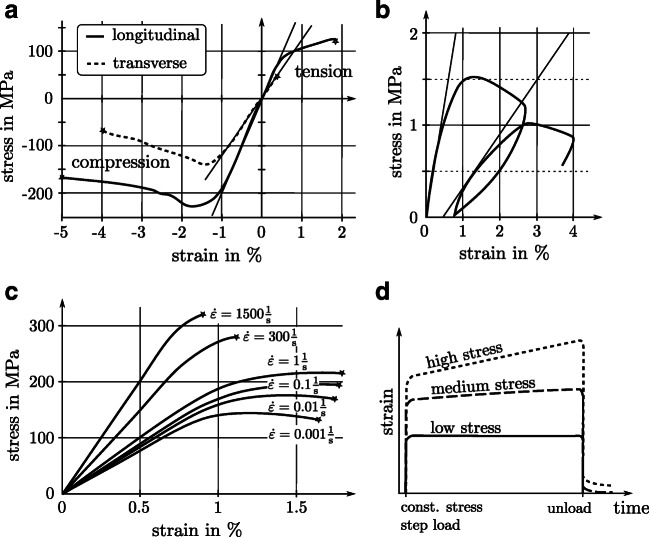
**a** Stress and strain of a quasi-static uniaxial tension/compression test on compact bone showing (quasi) linear region, yielding, and failure performed in the longitudinal and transverse direction. The figure was adapted from [24] and used with permission from Elsevier. **b** Uniaxial compressive cyclic behavior of trabecular bone showing yielding, stiffness reduction due to damage, and the remaining plastic deformation after unloading. The figure was adapted from [21] and used with permission from John Wiley and Sons. **c** The dependency of stiffness, yield, and failure on strain rate was obtained from compression tests on human cortical bone. The figure was adapted from [25] and used with permission from the American Physiological Society. **d** Creep behavior of cortical human bone. Three stress regimes are shown. For low stress, no creep occurs. For stress above a certain threshold, plastic creep strain is accumulating over time. The figure was adapted from [11, 41] and used with permission from Elsevier

When loading macroscopic bone specimens in a testing frame, they react elastically if the load does not exceed the yield limit. While there are no differences between tensile and compressive elastic stiffness for compression and tension [38], there are differences in yield stress, strength, and fracture strain, Fig. 2a [5, 13, 34]. Due to its microstructure, bone’s mechanical properties are direction dependent (i.e., anisotropic) but can be approximated with transverse isotropic or orthotropic material symmetry [9, 20, 33]. Consequently, when testing specimens from long bones in a longitudinal direction, a higher stiffness, yield stress, and strength can be observed, compared with testing in the transverse direction (Fig. 2a). Determining all 5 (transverse isotropy) or 9 (orthotropic) elasticity constants of bone experimentally is a very difficult task, for which ultrasonic measurements or a combination of tensile/torsion/shear tests can be used [54, 55].

When loading bone past the yield point, two energy dissipation mechanisms occur: plasticity and damage Fig. 2b. Plasticity is manifest as permanent deformation after unloading, whereas damage is associated with a reduction of stiffness due to the formation of microcracks [21, 44, 47]. In contrast, the appearance of macroscopic cracks is denoted as a *fracture*. At very high compressive strains, trabeculae collapse and *densification* appear which leads to rising stresses.

Bone mechanical behavior is influenced by viscosity and is therefore time dependent. More precisely, its stiffness, yield limit, and strength depend on the strain rate. These quantities are larger, the higher the speed at which load is applied. At the same time, at very high strain rates, the bone becomes less ductile and the fracture strain is reduced (Fig. 2c) [8, 18, 25]. Moreover, bone also exhibits creep—the accumulation of plastic deformation under constant load—provided that the load exceeds a certain threshold (Fig. 2d).

These mechanisms of elasticity, plasticity, damage, viscosity, and creep are constitutive mechanical properties of the material bone and intrinsic in nature. They are always present but appear in different accentuations depending on the hydration state, the molecular composition, and the microstructure of the respective bone specimen. For example, dry bone tissue is more brittle and less viscous than bone with physiological water content.

**Fig. 3:**
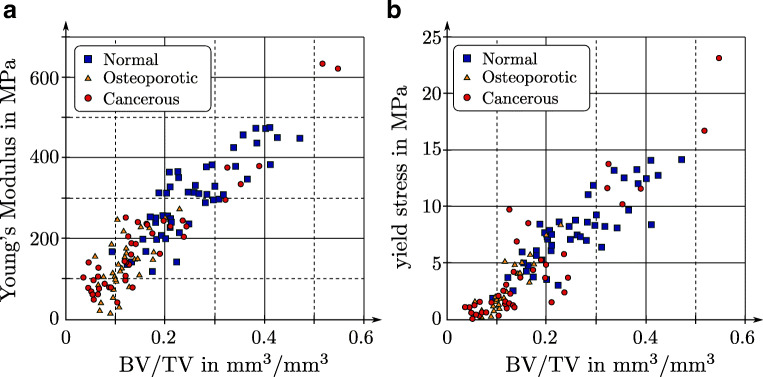
Dependency of stiffness **a** and yield stress **b** of human trabecular bone on BV/TV (bone volume/tissue volume). Healthy, osteoporotic, and cancerous BV/TV regimes are indicated. Adapted from [27], with permission from Springer Nature

Furthermore, it is well known that the apparent stiffness, yield stress, and strength are reduced when porosity increases. This is evident for pathologic bone alterations like osteoporosis or cancer, in which the bone mass is reduced and subsequently, porosity is increased compared with healthy bone [4, 15, 48, 52]. Figure 3 shows the relationship between BV/TV (the fraction of actual bone volume in a RVE) of trabecular bone specimens and stiffness as well as yield stress. Both properties are severely reduced for osteoporotic and cancerous BV/TV values [27]. Although not clearly visible in Fig. 3, usually, a nonlinear relationship exists between material parameters and BV/TV. It is known as a “power law density-elasticity relationship” that has usually the form

$$ E=A\ {\left(\frac{BV}{TV}\right)}^B $$

with *E* being the elastic modulus and *A* and *B* being constants [56–58].

### The Principle of Constitutive Modeling

**Fig. 4:**
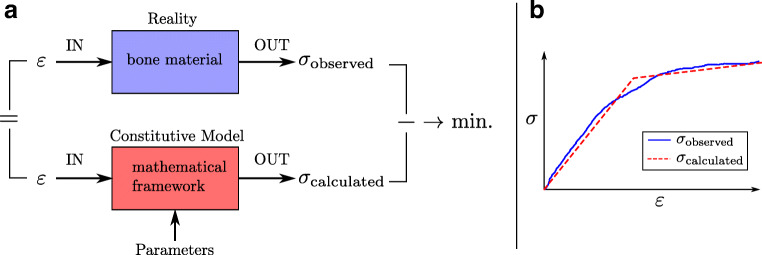
**a** Principle of constitutive modeling. Material behavior is observed in reality by subjecting the material to an input stimulus (e.g., strain) and observing the output (e.g., stress). By creating a constitutive model, it is attempted to obtain a similar output for the same input and—at the same time—obtain trustworthy outputs for new input stimuli within a certain range of applicability. Parameters are used to tune the model to a certain type of material. **b** Stress-strain curves illustrating an exemplary experimentally observed behavior *σ*_observed_ and the calculated model behavior *σ*_calculated_. Here, an elasto-plastic model is shown with a fair match of the observed data

A constitutive material model is aiming to replicate what has been observed in reality by means of a mathematical framework based on fundamental physical principles (Fig. 4). In addition to replicating, it should be predictive, which means that although being derived from a cloud of discrete sample data, the model should be able to calculate responses that have not been tested experimentally. This allows for its use for, e.g., implant design or clinical evaluation of bone strength.

In the sense of mimicking a material response to a mechanical stimulus, a constitutive material model is operating in the scheme of stress and strain. It attempts to return a stress output similar to the real bone material when subjecting it to a certain strain input stimulus (or vice versa) (Fig. 4).

A constitutive model as such generally captures certain classes of constitutive behavior, e.g., elasticity, plasticity, and damage as described in “The Biological Material “Bone”” section. To get a good match with the individual material, a number of model parameters must be adapted accordingly (Fig. 4b). Those parameters can be (i) material parameters like Young’s modulus or yield stress, (ii) microstructural parameters like BV/TV or degree of anisotropy, or (iii) parameters with no physical meaning like exponents in power laws.

Before applying a new constitutive model to a practical problem, it needs to be properly validated. By *validation*, it is confirmed that the model is performing as expected and its output is in acceptable accordance with real-world observations. Usually, model validation is performed either in a dedicated set of experiments or with respect to another already validated model. Due to experimental restrictions, a validation might not be carried out on the length scale of the model but on a higher length scale, which is introducing additional uncertainties.

Considering the complex structure and behavior of bone tissue from “The Biological Material “Bone”” and “Mechanical Behavior of Bone” sections, constitutive material models for bone can be classified according to (a) *length scale* (RVE size, i.e., considered structure like cortical bone, trabecular bone, single trabeculae, homogeneous, or heterogeneous), (b) *material symmetry* (e.g., isotropic, orthotropic), (c) *loading capabilities* (small or large strain amplitudes, monotonic or cyclic amplitudes, low or high strain rates), and (d) *constitutive response* (elastic, plastic, viscous, damage, fracture, densification).

Constitutive models that cover a wide *predictive range* of material behavior are difficult to formulate mathematically and even more difficult to validate experimentally. Thus, constitutive models are formulated for a certain range of applications in which they describe reality within an accepted accuracy. If used outside those limits, the model will return data which is then deviating from what would be observed in an experiment more than can be accepted. For example, a simple linear elastic material model should not be used for strains that exceed the elastic limit of the material.

## Review of Recent Constitutive Models

The second part of this review deals with the question if improved or new constitutive models have been developed and to what extent these findings influence bone strength prediction or related problems in musculoskeletal research.

### Literature Search

A systematic literature search was done on PubMed. The intention was to review recent advances with respect to constitutive bone models. Thus, the search was limited to the past 5 years. An overview of constitutive models prior to 2016 can be found in [40•]. Studies were excluded if they did not present a reasonable new or improved constitutive model for bone.

### Results

Selected publications on constitutive modeling of various types of bone are summarized in Table 1 and associated validations (experimental or numerical) are presented in Table 2. In the following, individual aspects will be reviewed in more detail.

**Table 1:** Overview of constitutive models for bone (past 5 years)

Author(s)	Year	RVE	Symmetry	Viscosity	Plasticity	Damage	Viscosity	Fracture	Densification
Schwiedrzik [43]	2016	tb-ti	iso	DPY(Q)	✓				
Panyasantisuk [32]	2016	tb-bo	ortho	DPY(Q)	✓				
Baumann [1]	2016	tb-ti	iso	PSD		✓			
		tb-ti	iso	DPY, DLY	✓				
Ng [28]	2017	ct-bo	iso	LFD	✓	✓			
Zysset [53•]	2017	ct-bo	N/A	1DY	✓	✓			
Ojanen [30]	2017	tb-ti	iso	BM			✓		
		tb-bo	iso	BM			✓		
Sabet [40•]	2018	tb-ti	iso	VMY, CIY, DPY	✓				
Haider [16]	2018	tb-bo	iso	BD		✓			
Ovesy [31]	2018	tb-bo	ortho	DPY(Q)+D	✓	✓			✓
Mirzaei [26]	2018	ct-bo	ortho	CZM				✓	
Werner [51]	2019	tb-ti	iso	VMY+C+D	✓			✓	
Shen [45]	2019	tb-bo	iso	PFM				✓	
Stipsitz [46]	2019	tb-ti	iso	DPD+D		✓		✓	
Lei [23•]	2020	ct-bo	trans	GMM			✓		
Reisinger [36]	2020	tb-ti	N/A	1DY	✓		✓		

*tb-bo*, trabecular bone Fig. 1b; *tb-ti*, trabecular tissue Fig. 1c; *ct-bo*, cortical bone Fig. 1d; *iso*, isotropic; *ortho*, orthotropic; *trans*, transverse iso; *N/A*, not applicable; *DPY(Q)*, Drucker-Prager yield, quadric approx. [42]; *DPY*, Drucker-Prager yield; *DLY*, Drucker-Lode yield; *PSD*, principle strain damage [29]; *LFD*, Lee-Fenves plastic damage [22]; *1DY*, one-dimensional yield; *BM*, Burger model; *VMY*, von Mises yield; *VM+C+D*, von Mises yield+cap+element deletion; *CIY*, cast iron yield; *BD*, Brittle damage [17]; *DPY(Q)+D*, DPY(Q)+densification [19]; *CZM*, cohesive zone model; *PFM*, phase-field model; DPD+D, DP+Q damage onset+element deletion; *GMM*, generalized Maxwell model

**Table 2:** Applications of constitutive models from Table 1. All reviewed papers contained either an experimental validation or numerical comparisons

Author(s)	Sample	Size (mm)	Load	Donor	Location	Validation	Sample no.	Software
Schwiedrzik [43]	tb cube	5	mono	hum	f, r, v	Exp	21	Abaqus
	tb cube	5	mono	hum	f, r, v	Exp	21	Feap
Panyasantisuk [32]	tb cube	5	mono	hum	f	Num	167	Feap
Baumann [1]	tb cube	5	mono	hum	f, v	Num	10	Custom
	tb cube	5	mono	hum	f, v	Num	10	Adina
Ng [28]	ct cube	*l*, *h* = 6, 8	mono	bov	f	Exp	6	Abaqus
	ct cylin	*d*, *h* = 4, 6	mono	bov	f	Exp	6	Abaqus
Zysset [53•]	ct cylin	*d*, *h* = 3, 6	cycl	bov	f	Exp	1^a^	Custom
Ojanen [30]	tb		mono	hum	f	Exp	11	Abaqus
	tb cylin	*d*, *h* = 10, 6	mono	hum	f	Exp	10	Abaqus
Sabet [40•]	tb cylin	*d*, *h* = 4, 8	mono	por	f	Exp	4	Abaqus
Haider [16]	femur		mono	hum	f	Exp	6	Abaqus
Ovesy [31]	tb cylin	*d*, *h* = 13, 19	cycl	hum	v	Exp	55	Abaqus
Mirzaei [26]	ASTM	*l* = 10 .*..* 50	mono	hum	f	Exp	8	Abaqus
Mirzaei [26]	femur		mono	hum	f	Exp	15	Abaqus
Werner [51]	tb cube	5	cycl	hum	f, v	Num	10	Abaqus
Shen [45]	humerus		mono	hum	h	Exp	1	Feap
Stipsitz [46]	tb cube	5	mono	hum	f, r, v	Exp	21	ParOSol^b^
Lei [23•]	ct cylin	*d*, *h* = 8, 7	mono	bov	f	Exp	60	Abaqus
Reisinger [36]	tb		cycl	hum	f	Exp	15	Custom

*tb*, trabecular/trabeculae; *ct*, cortical; *d, h, l*, diameter, height, length; *mono*, monotonic; *cycl*, cyclic; *cylin*, cylinder; *ASTM*, ASTM tensile and bending; *f*, femur; *r*, radius; *v*, vertebra; *h*, humerus; *hum*, human; *bov*, bovine; por, porcine; *Exp*, experimental; *Num*, numerical

^a^Qualitative comparison

^b^Nonlinear extension

#### Predictive Range of Models

Most models include plasticity, damage, and/or fracture. Viscosity and densification are rarely implemented. Usually, the models cover a specific range of predictable constitutive behavior.

#### New Developments

Zysset et al. [53•] presented a new development regarding in-elastic material behavior via microcrack opening and closing. Qualitatively, the results fitted well with cyclic experiments but the model is currently limited to 1D and cortical bone. This mechanically intuitive constitutive model is able to describe the loading and unloading of bone very realistically, forms the basis for improved constitutive model developments, and extents future research possibilities. Also, Reisinger et al. [36] presented a new 1D model for bone tissue combining plasticity and viscosity, validated on single bone trabeculae. This model includes viscosity and is fitted based on novel experiments at a single trabeculae level under wet conditions.

#### Elastic Limit/Plasticity

A common challenge is to define the onset of elasticity in the case of multi-axial stresses. *Yield surfaces* based on Drucker-Prager, von Mises, or principle strain are typically used. Specifically, in the reviewed works, the elasticity limit is mostly based on a quadratic criterion of Drucker-Prager type and the post-yield behavior includes linear isotropic hardening. Some authors applied theories from other engineering areas such as the Lee-Fenves model from civil engineering [28], phase-field models from material engineering [45], or cohesive zone models from composite engineering [26]. Only the latter study provided a reasonable high number of experiments and showed fracture load correlations of *R*^2^ = 0*.*89 for the presented cohesive zone model. In general, the multi-axial experimental validation of yield surfaces requires bi- or triaxial testing equipment and is thus very challenging [*F*, *G*].

#### Micro-architecture

On the apparent trabecular bone level (Fig. 1b), architectural features like BV/TV and anisotropy (DA) are still the gold standard independent parameters to predict apparent stiffness and strength based on these CT-based measures [31, 32] and two study approaches were found: first, direct homogenization of the apparent level by using bone density (power laws [16, 45]) and, in some cases, the orientation of the trabecular network (fabric [31, 32]). Power laws are simple to implement and are the choice if only bone density is available, whereas orthotropic fabric-based models are more accurate but such models need information about the trabecular bone orientation. In the second case, trabecular bone micro-FE models were used together with local constitutive modeling at the tissue level. However, such modeling requires high-resolution imaging as input and a direct validation was not done. Cortical bone was considered as homogeneous, isotropic, or orthotropic material and microstructures like pores are not considered explicitly.

#### Loading

Locomotion or movements are associated with dynamic loading of the human skeleton. Although constitutive models should in principle be able to model both loading and unloading, only Zysset and his team [31, 51, 53•] and Reisinger et al. [36] compared their models to cyclic loading-unloading protocols. All others examined only monotonically increasing loads and, therefore, do not allow any conclusions to be drawn about the correct unloading behavior of the model. In the case of clinical problems, whenever cyclic loading appears usually studies are carrying out by lab experiments instead of computer modeling because of its complexity. At this point, such new material models presented here could make a notable contribution. With respect to the loading speed, all models are based on one speed except for the work of Lei [23•].

#### Damage and Fracture

Interestingly, models including fracture mechanics became increasingly popular in the last years. Either rather simple “element deletion” models [46, 51] or more sophisticated fracture energy-based models [26, 45] have been published. Such approaches are more and more used in engineering fields and are available in commercial software packages which makes their use relatively easy. However, local “crack” modeling strategies include a process zone or mesh dependency at high strain amplitudes. This introduces a further—sometimes invisible—tuning parameter. Put simply, the in-elastic modeling effort is shifted from the development of a sophisticated constitutive model (including such processes) to a numerically more expensive simulation model which gives a good insight into the progression of fracture processes.

#### Anatomical Samples

Material laws based on experimental studies provide parameters that depend on the biological material used but also on the shape of the sample and other factors like the number of samples and applied loading. Many studies used human femora bone but also studies on animal bones (bovine, porcine) were published for experimental comparison. Some studies were based on existing experimental results. As usual in the case of constitutive developments, many experiments (Table 2) were carried out on trabecular and cortical cubes/cylinders with a size of approx. 3–20 mm or whole bone parts. Only a few studies presented a sufficiently high number of specimens to capture the anatomical variations.

#### Model Validation

To begin with, it should be mentioned that outcomes presented in Table 2 which are based on a few specimens should be rather seen as a proof-of-concept than a validation. A direct multi-axial validation of clinically applicable apparent bone models is currently not possible experimentally. Therefore, as mentioned above, CT-based micro-FE models are used together with computational homogenization to predict the apparent behavior. However, Baumann et al. [1] stated that it is unlikely that a tissue constitutive model can be fully validated from apparent level experiments alone, as calculations are too insensitive to identify differences in the outcomes. In this context, the results of Sabet et al. [40•] demonstrated that using three different tissue constitutive models had only a slight effect on the apparent response. As expected, there was a significant change in the apparent response which goes back to the bone volume fraction. In the case of viscosity, Ojanen et al. [30] found that bone tissue visco-elasticity may not fully be able to predict the macro-scale visco-elasticity in human trabecular bone.

#### Software

In order to use the developed constitutive laws for real bone models, they were implemented in finite element codes. Although many software packages enable nonlinear material modeling, only a few different packages are reported. Mainly, the commercial software Abaqus (Dassault Syst̀emes Simulia Corp, Providence, RI) is used. It allows for any nonlinearity and the possibility of user material modeling. Parallelizable codes like Feap (University of California, Berkeley) and ParOSol (ETH Zürich, Switzerland) also formed the basis for some studies which provide even more implementation flexibility and a better parallel execution performance [46].

#### Clinical Applicability

Most of the presented constitutive models can be used for bone strength assessment based on quantitative information about bone density. However, applicable new or sophisticated engineering approaches with respect to constitutive modeling of bone were not identified.

## Discussion

The mechanical response of bone depends on factors like the structure or length scale under consideration and the applied loading regime. Constitutive models try to describe this response through mathematical models. Usually, such models are implemented into finite element tools to allow the analysis of complex geometries, material distributions, and loading conditions.

The focus here was to evaluate recently published constitutive laws for trabecular or cortical bone with respect to practical usage, e.g., in CT-based bone strength predictions.

In summary, there are advances in the field of constitutive modeling of bone tissue. The main focus of recent studies was on the extension and experimental validation of existing models. There were a few new developments, but no fundamental improvements were found compared with the years before. Most important seems the consideration of physical mechanisms (e.g., microcracking) in the case of combined loading. In this regard, a generalization to 3D and application on bone structures which are subjected to cyclic overloading, e.g., bone implant systems, should be researched. Interestingly, a trend towards fracture-based models is observed over the last years. Such models put less effort into the local constitutive modeling but could lead to more insight into the overall failure mechanisms of bone. This might inspire new sophisticated constitutive model developments.

Some studies showed that a multi-scale validation (tissue to apparent level) is still inaccurate. Obviously, important features are missing or experimental shortcomings influence the results. For this reason, validations should take place on the same length scale on which the model is operating. For investigations at any level, more experimental data are desirable taking into account local strain measurements, multi-axial loading, and cyclic loading at different strain rates.

Model validations were carried out in vitro and showed similar accuracies as previous studies. One difficulty in recognizing the actual improvement through a new constitutive model lies in the fact that poor image quality and calibration or incorrect or oversimplified loading have a strong impact on the quality of the obtained results. This means there will be plenty of scope for further improvement of constitutive bone models, but at the same time, other uncertain model parameters must be reduced.

A general problem with simulation methods in biomechanics is the lack of standardization. Sample preparations, experimental protocols, tissue types, scanning, image processing, FEA modeling, and post-processing are done differently from study to study. It is not possible to compare constitutive models quantitatively making it impossible to separate good from less good models.

In terms of clinical applicability in osteoporosis research, in vivo CT-based FEA simulations based on sophisticated constitutive behavior still have great potential. Although no great achievements regarding constitutive modeling have been achieved, the FEA technology itself has developed accordingly. There is an increased level of automation and computational capabilities and the insights and predictive power of FEA simulations models are far better than estimates based on bone mass [49] or other non-physical image-based measures.
